# Audit of the ward-based management of severe sepsis in a large teaching hospital

**DOI:** 10.1186/cc11763

**Published:** 2012-11-14

**Authors:** G Estebanez, M Cole

**Affiliations:** 1 Royal Liverpool and Broadgreen University Hospitals NHS Trust, Liverpool, UK

## Background

The Surviving Sepsis 6-hour bundle [1] was created to promote effective identification and management of patients with severe sepsis and septic shock. Efficient and timely implementation of the 6-hour bundle including early intravenous antibiotics has been shown to improve patient outcome [2]. We aimed to determine the efficiency of severe sepsis identification, implementation of the 6-hour bundle and overall management of ward-based patients.

## Methods

During a 4-month period, ward-based patients with severe sepsis were identified by nurse practitioners and the critical care outreach team at a large teaching hospital in North West England. Analysis of patient medical records was performed to assess the efficiency and quality of care received compared with the gold standard sepsis 6-hour bundle. Promptness of doctor attendance and patient 30-day outcome were also analysed.

## Results

Twenty-eight patients with severe sepsis were identified and analysed. Suspicion of sepsis was documented in medical records for all patients; however, the 6-hour sepsis bundle was completed in only one case. Appropriate i.v. antibiotics were prescribed to all patients. Median duration from the documented onset of severe sepsis (time 0) until review by a doctor was 90 minutes (0 to 19 hours). The median Modified Early Warning Score (MEWS), determined from patients' vital signs, at documented onset of severe sepsis was 3.5 (1 to 9) with a subsequent increase of median MEWS to 4 by the time of doctors' first attendance. The 30-day patient outcome showed 13 patients discharged home and seven patients deceased. Comparison of deceased patients with patients who were discharged alive (see Table 1) demonstrated an increased median duration from time 0 until doctor attendance in the deceased group. However, interestingly, timely antibiotics (given within 1 hour of patient assessment by a doctor) were administered with greater frequency in the deceased patient group.

**Table 1:** Characteristics and management data for patients with severe sepsis comparing patients who were deceased with patients discharged home at 30 days

	Patients discharged home at 30 days (*n *= 13)	Patients deceased at 30 days (*n *= 7)
Median age (years)	77	68
Median time taken to be seen by doctor after time 0 (minutes)	90	240
Median MEWS at time 0	3	3
Median MEWS at doctors' attendance	3	4
Timely antibiotics given	15%	43%
Basic resuscitation undertaken (oxygen, i.v. fluids, urinary catheter, ABG)	90%	93%

Time 0 = time of initial documented evidence of severe sepsis.

## Conclusion

Despite the widespread recognition of patients with severe sepsis, underperformance of the 6-hour bundle remains a major factor in suboptimal management of ward-based patients. Increased delay from the onset of severe sepsis until doctor review is associated with increased risk of mortality despite better adherence to the 6-hour bundle. Further education of doctors and nursing staff, regarding the importance of the 6-hour sepsis bundle, in addition to the implementation of strategies to improve the early identification and timely review of ward-based patients with severe sepsis are recommended.
